# Altered brain–behavior coupling during inhibitory control in ankylosing spondylitis: ERP evidence from NoGo-P3 component

**DOI:** 10.1371/journal.pone.0351397

**Published:** 2026-06-12

**Authors:** Lei Zhang, Fang Lu, Yuxin He, Lin Tang, Li Zhang, Jing Xiang, Chun rong Gu, Su wan Guo, Zheng hong Yu

**Affiliations:** 1 Department of Rheumatology and Immunology, Jinling, Hospital, Affiliated Hospital of Medical School, Nanjing University, Nanjing, China; 2 School of Early-Childhood Education, NanJing XiaoZhuang University, Nanjing, China; 3 Software Institute, Nanjing University, Nanjing, China; 4 School of Health Policy and Management, Nanjing Medical University, Nanjing, China; 5 Binhai County People’s Hospital, Yanchen, China; 6 Department of Psychiatry, Affiliated Brain Hospital of Nanjing Medical University‌‌, Nanjing, China; Federal University Oye-Ekiti, NIGERIA

## Abstract

**Background:**

Cognitive dysfunction has increasingly been recognized in Ankylosing Spondylitis (AS), yet the neural mechanisms underlying inhibitory control in this population remain insufficiently characterized. Rather than reflecting a simple global deficit, cognitive alterations in AS may involve task-dependent changes in the coupling between neural activity and behavioral performance. This study examined executive control in AS using a Go/NoGo paradigm and focus on brain-behavior coupling during inhibitory processing.

**Methods:**

16 male patients and 23 age-matched healthy controls completed a Go/NoGo task while undergoing 32-channel EEG recording. ERP analyses focused on N2 (200–300ms) and the late positive component in the NoGo condition (hereafter termed NoGo-P3; 400–600ms). Mean amplitudes were extracted at fronto-parietal midline electrodes. To directly test group differences in brain–behavior coupling, linear regression models including the ERP × Group interaction term were fitted. Theta-band (4–7 Hz) power within the 400–600 ms window was additionally analyzed using FFT-based spectral estimation.

**Results:**

Behaviorally, AS patients showed lower Go accuracy and longer Go reaction times, together with a tendency toward higher NoGo accuracy. In AS patients, NoGo accuracy positively correlated with NoGo-P3 amplitude at FCz (r = .64, p = .009) and Cz (r = .55, p = .035), whereas these associations were not significant in controls. Direct group comparison showed a significant ERP × Group interaction at FCz (b = 0.084, p = 0.033), indicating that the relationship between NoGo-P3 amplitude and inhibitory accuracy differed between AS patients and healthy controls, while Cz (b = 0.051, p = 0.106) showed a similar but non-significant trend. Complementary theta analyses revealed enhanced post-stimulus theta power in centro-parietal regions during NoGo processing in AS.

**Conclusions:**

The findings suggest altered brain–behavior coupling during inhibitory control in AS, with the most robust evidence emerging from the NoGo-P3 component at FCz. This pattern is consistent with greater reliance on effortful control-related neural recruitment during successful inhibition and may represent a candidate electrophysiological marker of altered executive processing in AS.

## Introduction

Ankylosing spondylitis (AS) is a chronic systemic inflammatory disease primarily affecting the sacroiliac joints and axial skeleton, and is typically characterized by pain, stiffness, and progressive functional limitation. In recent years, increasing attention has been directed toward the potential impact of chronic inflammatory states on the central nervous system and cognitive functioning [1–3]. Related research has moved beyond simply asking whether inflammation affects brain function and has begun to explore the possible neuroimmune pathways and brain alterations involved [2,3]. Existing evidence suggests that persistent inflammatory activity may be associated with abnormal neurotransmitter regulation, reduced neural processing efficiency, and impaired integration of large-scale brain networks [4–6]. Against this background, cognitive changes in chronic inflammatory diseases have gradually become a clinical issue of growing concern; in rheumatology, cognitive impairment has also begun to be regarded as a clinically meaningful dimension of disease burden, rather than merely a secondary consequence of pain or fatigue [7].

Among the cognitive domains potentially affected in AS, executive function is particularly important because it supports adaptive, goal-directed behavior under changing internal and external demands. Executive control includes processes such as monitoring, updating, planning, and response inhibition, all of which are essential for behavioral flexibility [8]. Inhibitory control is especially relevant because it enables the suppression of prepotent but inappropriate responses and is highly sensitive to the integrity of frontal control systems [9–11]. In AS, chronic pain, fatigue, sleep disturbance, and the cumulative burden of persistent inflammatory disease may together increase cognitive load and reduce the efficiency of control-related neural systems [12–18]. Recent evidence from inflammatory rheumatic disease populations further suggests that cognitive outcomes may be shaped not only by disease status itself, but also by broader clinical burden, including affective and functional factors [19]. These considerations make inhibitory control a theoretically and clinically meaningful entry point for studying cognition in AS.

The Go/NoGo paradigm is a well-established experimental model for investigating inhibitory control. In this task, frequent Go trials create a strong tendency to respond, whereas infrequent NoGo trials require participants to suppress an already prepared motor response. This contrast makes the paradigm particularly suitable for examining the temporal dynamics of response inhibition and control allocation. Importantly, task structure itself can influence inhibitory processing, and the relative proportion of Go and NoGo trials has been shown to modulate both behavioral performance and inhibition-related ERP activity [20]. In the present study, a 4:1 Go-to-NoGo ratio was used to establish a prepotent response tendency and thereby increase inhibitory demand on NoGo trials.

Event-related potentials (ERPs) provide temporally precise indices of inhibitory processing during Go/NoGo performance. Two components have been especially relevant in this literature: the N2 and the later positive component observed during successful inhibition [21–25]. The N2, typically examined in the 200–300 ms latency range, has often been associated with early conflict monitoring and the detection of competing response tendencies [21]. However, its functional significance is not entirely uniform across studies, and its magnitude may vary depending on task structure, context, and electrode site. By contrast, the later positive component during NoGo trials has more consistently been linked to later-stage inhibitory evaluation, response monitoring, and the allocation of control-related resources [22,23,25]. More recent ERP work has likewise emphasized that the later P3-related response may be more closely tied than N2 to evaluative and decision-related aspects of inhibitory processing, particularly under NoGo demands [26]. For this reason, the NoGo-related late positive component is of particular interest in the present study.

In addition to time-domain ERP indices, oscillatory activity in the theta band has frequently been implicated in executive control. Theta activity, especially over frontal and midline regions, has been associated with conflict monitoring, control engagement, and adaptive behavioral regulation [24,27–29]. In inhibitory tasks, increased theta power is often interpreted as reflecting greater recruitment of control-related neural resources. At the same time, oscillatory findings in clinical populations should be interpreted cautiously, particularly when detailed disease-activity and biomarker information are not comprehensively available. In the present study, theta activity was therefore treated as a complementary neural index that could help contextualize ERP findings, rather than as a standalone mechanistic marker.

Although prior work suggests that AS may be associated with executive inefficiency, the neural processes underlying inhibitory control in this condition remain insufficiently characterized. Most existing studies have emphasized broad cognitive outcomes or group differences in mean behavioral and neural responses. Such approaches are informative but may overlook a more functionally meaningful possibility: patients and controls may achieve superficially similar task performance through different patterns of neural recruitment. In other words, the most informative alteration may lie not only in whether a neural response is larger or smaller on average, but in how strongly that neural response is linked to successful performance.

For this reason, the present study places particular emphasis on brain–behavior coupling. Rather than assuming that cognitive alteration in AS must manifest as a simple global deficit, we tested whether the relationship between inhibitory-control-related neural activity and behavioral performance differs between patients with AS and healthy controls. This approach allows a more functionally informative interpretation of the data by examining whether neural signals are differentially associated with successful inhibition across groups.

To address this question, we employed a Go/NoGo paradigm combined with EEG recording in male patients with AS and age-matched healthy controls. We examined behavioral performance, ERP mean amplitudes in predefined latency windows, and theta-band power during inhibitory processing. ERP analyses focused on the N2 (200–300 ms) and the NoGo-P3 (400–600 ms), with particular attention to fronto-central and centro-parietal midline electrodes commonly implicated in inhibitory control. In addition, we tested whether NoGo accuracy was differentially associated with NoGo-P3 amplitude across groups.

Based on prior work on inhibitory control and cognitive adaptation under clinical burden, we expected that AS patients would show altered neural recruitment during inhibition, even if overt behavioral deficits were not uniformly pronounced. More specifically, we hypothesized that the association between NoGo-P3 amplitude and inhibitory accuracy would be stronger in the AS group than in healthy controls, indicating altered brain–behavior coupling during successful response inhibition. The N2 was included as an additional index of early inhibitory processing, but our primary interest concerned the later-stage inhibitory component and its relationship to behavioral performance.

## Materials and methods

### Participants

A total of 39 male participants were included in the present study, comprising 16 patients with AS and 23 age-matched healthy controls; no significant between-group differences in age were observed. All AS patients were diagnosed by board-certified rheumatologists at a tertiary care hospital and were in a stable disease state without acute flare-ups during testing. Group classification in the present study was therefore based on clinical diagnosis rather than detailed disease-activity or biomarker profiling. None of the participants had a history of psychotropic medication use, and all participants were right-handed. Participants were recruited up to August 2025, and the present analyses were based on the final sample obtained within this recruitment period. A total of 41 individuals were intially enrolled, of whom 39 met prespecified quality-control and eligibility criteria and were included in the analyses. All participants provided written informed consent prior to participation. The study protocol and consent procedures were approved by the Scientific Research Ethics Committee of Nanjing Xiaozhuang University (Ref. XZ2025NO.2025110; approval date: June 3, 2025) and adhered to the ethical standards of the Declaration of Helsinki.

### Experimental task and procedure

A classic Go/NoGo task paradigm was employed to assess inhibitory control [21–23]. The Go stimulus was the letter “W,” and the NoGo stimulus was the letter “M.” Participants were instructed to press the “J” key as quickly and accurately as possible in response to the Go stimulus and to withhold their response when the NoGo stimulus appeared. Each stimulus was presented centrally for 200 ms, and the inter-stimulus interval (ISI) was fixed at 1000 ms. The experiment consisted of 150 trials, with a Go to NoGo ratio of 4:1 to establish a prepotent response tendency and thereby increase the inhibitory demands on NoGo trials [20]. Before the formal experiment, participants completed practice trials and proceeded to the experimental session only after achieving 100% accuracy. The stimulus sequence was randomized, and no trial-by-trial feedback was provided during the formal task.

### Behavioral data acquisition and processing‌‌

Behavioral indices included Go-trial accuracy (Go_ACC), Go-trial reaction time (Go_RT), NoGo-trial accuracy (NoGo_ACC), and commission-error reaction time in the NoGo condition (NoGoError_RT). Trials with reaction times shorter than 200 ms or longer than 1000 ms were excluded from analysis as outliers. Between-group comparisons of behavioral performance were conducted using independent samples t-tests. Descriptive statistics for behavioral variables are reported as mean ± standard deviation (SD) for each group. All behavioral data processing and statistical analyses were performed in MATLAB R2024b.

### EEG data acquisition and preprocessing

Continuous EEG data were recorded using a 32-channel NeuroScan system (Compumedics NeuroScan, USA) at a sampling rate of 1000 Hz. Electrodes were placed according to the international 10–20 system, with linked mastoids serving as reference electrodes. Electrode impedances were maintained below 5 kΩ throughout the recording session.

Offline preprocessing was performed in EEGLAB (version 2023.01) within the MATLAB environment. EEG signals were band-pass filtered between 0.1 and 30 Hz. Independent Component Analysis (ICA) was applied to identify and remove ocular artifacts. The continuous data were then segmented into epochs extending from −200–1000 ms relative to stimulus onset. Epochs exhibiting voltage fluctuations exceeding ±100 μV were excluded from further analysis.

To improve transparency regarding data quality, accepted trial counts after preprocessing were summarized separately for Go and NoGo trials in each group (A summary of accepted epoch counts by group and condition is provided in Supplementary S1 Table). After preprocessing, the mean number of accepted Go epochs was 115.63 ± 9.14 (range:84–120) in the AS group and 119.17 ± 0.83 (range:117–120) in the control group. For NoGo trials, the mean number of accepted epochs was 15.80 ± 9.41 (range:4–30) in the AS group and 24.17 ± 6.15 (range:9–30) in the control group. One patient was excluded from all NoGo ERP–behavior analyses because no artifact-free NoGo trials remained after preprocessing.

### ERP component analysis

ERP analyses focused on two predefined latency windows: the N2 (200–300 ms) and the later positive component in the NoGo condition, hereafter termed the NoGo-P3 (400–600 ms) [21,22,25,26]. These time windows were defined on the basis of prior Go/NoGo literature and visual inspection of the grand-average waveforms. Mean amplitudes were extracted at five fronto-parietal midline electrodes: Fz, FCz, Cz, CPz, and Pz. These sites were selected because they are commonly implicated in inhibitory control and are consistent with prior Go/NoGo ERP studies [21–25].

For each participant and task condition, ERP mean amplitude was calculated within the predefined latency window at each electrode. N2 analyses were treated as an index of early conflict-related processing, whereas the NoGo-P3 was treated as the principal later-stage index of inhibitory evaluation and control-related resource allocation. Both components were quantified as mean amplitudes within predefined latency windows, rather than as individual peak amplitudes. Accordingly, although the waveform could show a negative-going deflection within the N2 latency range, the window-averaged values were not necessarily negative across all electrodes and conditions. Because the later NoGo-related positive component in the present study was examined under inhibitory demands and showed a fronto-central to centro-parietal distribution, it is referred to throughout the revised manuscript as the NoGo-P3 rather than canonical P3b.

### Time-frequency analysis

To complement the ERP analysis, theta-band power was examined as an additional neural index of control-related processing [24,27–29]. Theta power was computed in the 400–600 ms post-stimulus window using FFT-based spectral estimation. EEG data were sampled at 1000 Hz, and spectral estimates were calculated using an NFFT of 1024. For each artifact-free epoch, mean power in the theta band (4–7 Hz) was computed and then averaged across epochs to obtain a participant-level theta estimate.

Theta analyses were first computed across 26 scalp electrodes (Fp1, Fp2, F7, F3, Fz, F4, F8, FC3, FCz, FC4, T7, C3, Cz, C4, T8, CP3, CPz, CP4, P7, P3, Pz, P4, P8, O1, Oz, and O2). The primary analyses focused on five a priori electrodes of theoretical interest (Fz, FCz, Cz, CPz, and Pz), consistent with the ERP analyses, whereas broader 26-channel topographical comparisons were treated as exploratory and are reported in the Supplementary Material. No additional baseline normalization was applied to the theta power estimates beyond the standard preprocessing baseline correction described above.

### Statistical analysis

All statistical analyses were conducted separately for the Go and NoGo conditions. Independent-samples t-tests were used to assess group differences in behavioral performance, ERP mean amplitudes, and theta power. For ERP analyses, the FCz and Cz NoGo-P3 measures were treated as a priori region-of-interest (ROI) analyses because these electrodes were central to the study’s main hypothesis regarding inhibitory brain–behavior coupling. Broader electrode-wise and topographical analyses were treated as exploratory.

To evaluate brain–behavior coupling, Pearson correlation analyses were first conducted within each group to examine the relationship between neural activity and behavioral performance. For Go trials, both accuracy and reaction time were examined; for NoGo trials, analyses focused on accuracy. Because significance in one group and non-significance in another does not by itself establish a between-group difference, we additionally fitted linear regression models including the ERP × Group interaction term to directly test whether the association between NoGo-P3 amplitude and NoGo accuracy differed between groups. One patient was excluded from these NoGo ERP–behavior analyses because no artifact-free NoGo trials remained after preprocessing.

For exploratory theta topographical analyses, group differences were assessed separately at each channel, and Bonferroni correction was applied across the tested channels. Bonferroni-corrected channel-wise results for the Go and NoGo conditions are reported in the Supplementary Material. All statistical tests were two-tailed, with significance set at p < .05 unless otherwise specified. Comprehensive supplementary tables provide the full set of ERP–behavior correlation results and the corrected exploratory theta results.

## Results

### Behavioral results

**Fig 1:**
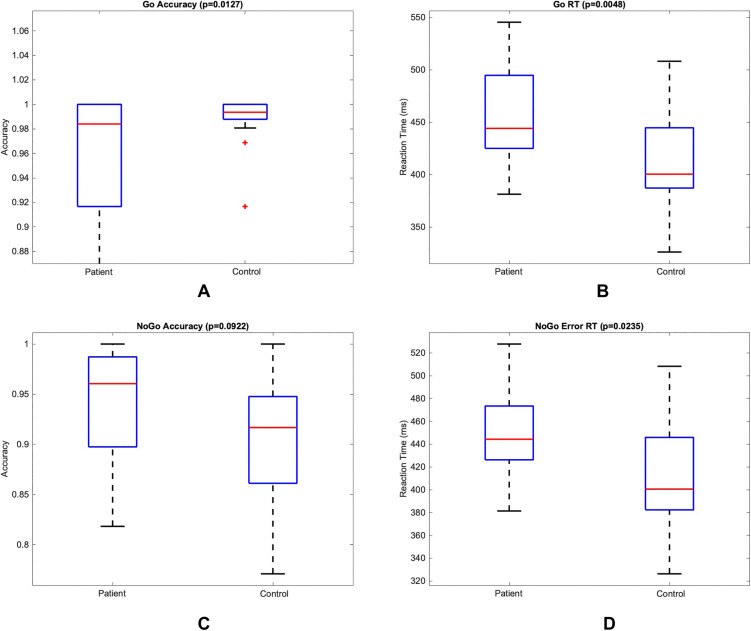
Go/NoGo task: Group differences in accuracy and reaction time. (A) Go Accuracy. The control group showed higher accuracy than the patient group (p = 0.0127). (B) Go Reaction Time (Go RT). The patient group showed longer reaction times than the control group (p = 0.0048). (C) NoGo Accuracy. No significant group difference was found (p = 0.0922). (D) NoGo Error RT. The patient group showed longer reaction times than the control group (p = 0.0235). The central red line indicates the median, the box represents the interquartile range (IQR, 25th to 75th percentiles), and the whiskers extend to the most extreme data points not considered outliers. Outliers are plotted individually as red crosses.

Behavioral performance differed between groups in a pattern suggestive of reduced response efficiency but relatively preserved inhibitory performance in the AS group (Table 1). Under the Go condition, patients with AS showed significantly lower accuracy than healthy controls (0.956 ± 0.050 vs. 0.987 ± 0.024; t(37) = −2.62, p = 0.0127; Fig 1A) and significantly longer reaction times (456.59 ± 48.01 ms vs. 410.49 ± 46.70 ms; t(37) = 3.00, p = 0.0048; Fig 1B), indicating reduced efficiency during response execution. Under the NoGo condition, the AS group showed numerically higher accuracy than controls (0.939 ± 0.058 vs. 0.905 ± 0.062; t(37) = 1.73, p = 0.0922; Fig 1C), although this difference did not reach statistical significance. In addition, NoGo error reaction time was significantly longer in the AS group than in controls (t(31) = 2.38, p = 0.0235; Fig 1D), suggesting that inhibitory performance was at least preserved and may have been achieved with a more cautious response style. Together, these findings suggest reduced response efficiency during Go performance, whereas under the NoGo condition, inhibitory accuracy was at least maintained and was accompanied by slower error responses in the AS group..

**Table 1:** Behavioral performance in AS patients and healthy controls.

Measure	AS (mean ± SD)	Control (mean ± SD)
**Go_ACC**	0.956 ± 0.050	0.987 ± 0.024
**Go_RT (ms)**	456.59 ± 48.01	410.49 ± 46.70
**NoGo_ACC**	0.939 ± 0.058	0.905 ± 0.062

### ERP results

#### Group differences under the go condition.

**Fig 2:**
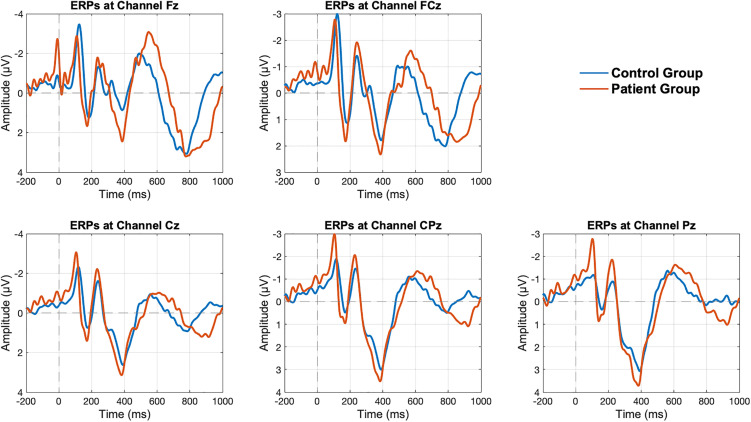
ERP waveform comparisons under the Go Condition across Five Midline Electrodes.

**Fig 3:**
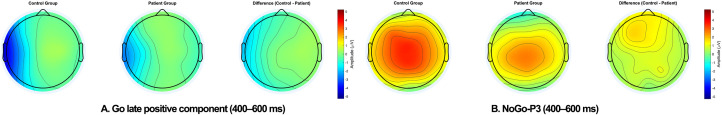
Scalp topographies of the late positive ERP component in the Go condition and the NoGo-P3 component in the NoGo condition (400–600 ms). (A) Scalp topographies of the late positive component in the Go condition. (B) Scalp‌‌ topographies of the NoGo-P3 component in the NoGo condition. For each panel, scalp maps are shown for the control group, the AS group, and the difference map (control−patient). A unified color scale was used within each panel to facilitate visual comparison.

Under the Go condition, both groups displayed the expected sequence of an early negative-going component within the N2 time window and a later positive-going activity within the predefined late time window. However, between-group differences in mean ERP amplitudes under the Go condition were limited. In the predefined 200–300 ms window, N2-related activity did not show robust group differences across the midline electrodes examined. Likewise, group differences in the later positive component under the Go condition were modest and did not constitute a central finding of the study. Overall, the ERP results under Go demands suggest that although behavioral execution was slowed in AS patients, the corresponding group differences in mean ERP amplitudes during simple response execution were not pronounced (see Supplementary S2 Table, Fig 2). As shown in the scalp topographies of the late positive component in the Go condition (see Fig 3A), the overall distribution was relatively diffuse and did not suggest a robust group difference.

#### Group differences under the NoGo condition.

Under the NoGo condition, the later positive component (NoGo-P3) showed a clearer and more theoretically relevant pattern than the N2. In the 200–300 ms window, N2-related activity was present but did not show a robust and stable group difference across the examined electrodes. Because N2 was quantified as mean amplitude within a predefined latency window rather than as individual peak negativity, its values were not uniformly negative across all electrodes and conditions, and its interpretation should therefore be cautious.

**Fig 4:**
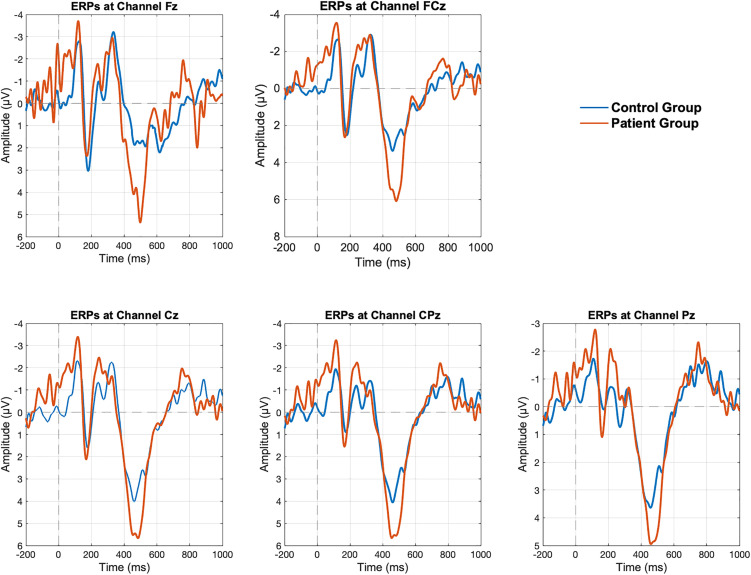
ERP waveform comparisons under the NoGo condition across five midline electrodes.

By contrast, the NoGo-P3 showed a more coherent distribution and a clearer relation to inhibitory performance. As shown in the scalp topographies of the NoGo-P3 (see Fig 3B), the NoGo condition exhibited a prominent fronto-central to centro-parietal distribution, consistent with the expected morphology of a NoGo-related late positive component. Although mean group differences in NoGo-P3 amplitude alone were not uniformly strong across all electrodes, the overall spatial and temporal pattern, together with the grand-average waveforms (Fig 4), supported treating the NoGo-P3 as the more functionally informative ERP index in the present study (see Supplementary S3 Table).

### ERP–behavior correlation analysis

Because mean ERP amplitude differences alone may overlook functionally meaningful group differences, we next examined whether the relationship between inhibitory-control-related neural activity and behavioral performance differed across groups. Correlation analyses focused on NoGo accuracy and NoGo-P3 mean amplitude (see Supplementary S4 Table).

**Fig 5:**
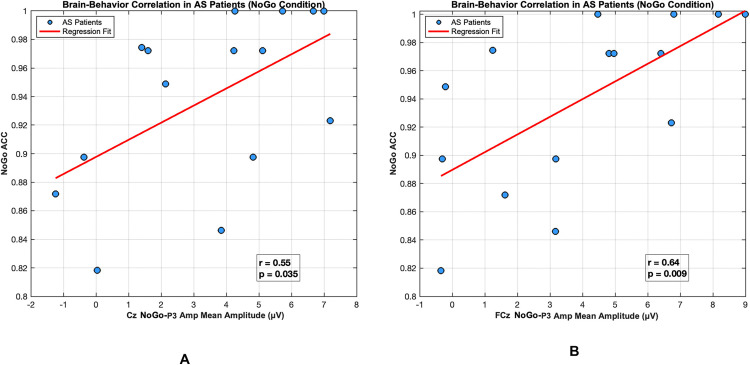
Positive associations between NoGo-P3 amplitude and NoGo accuracy in AS patients under the NoGo condition. (A) Scatter plot of mean NoGo-P3 amplitude at the Cz electrode against NoGo accuracy. (B) Scatter plot of mean NoGo-P3 amplitude at the FCz electrode against NoGo accuracy. Blue circles represent individual patient subjects, and the red solid line indicates the best-fit linear regression. The text box in the lower right corner displays the Pearson correlation coefficient (r) and the significance level (p).

In the AS group, NoGo accuracy was positively correlated with NoGo-P3 amplitude at FCz (r = 0.645, p = 0.009) and Cz (r = 0.546, p = 0.035) (see Fig 5). In contrast, these associations were not significant in the control group (FCz: r = 0.169, p = 0.441; Cz: r = 0.194, p = 0.376). To directly test whether the ERP–behavior association differed between groups, linear regression models including the ERP × Group interaction term were fitted. The interaction was significant for FCz (b = 0.084, p = 0.033), indicating that the association between NoGo-P3 amplitude and inhibitory accuracy differed between AS patients and healthy controls. The corresponding interaction for Cz showed a similar direction but did not reach statistical significance (b = 0.051, p = 0.106; see Supplementary S5 Table).

These findings suggest that the most robust group-dependent brain–behavior coupling effect was localized to FCz, while Cz showed a convergent but less stable pattern. Thus, the principal neural difference between groups was not simply reflected in average ERP amplitude, but rather in how strongly NoGo-P3 activity was linked to successful inhibition.

One patient was excluded from these NoGo ERP–behavior analyses because no artifact-free NoGo trials remained after preprocessing.

### Theta power analysis in the NoGo-P3 time window

Theta-band activity was analyzed in the 4–7 Hz range within the 400–600 ms post-stimulus window as a complementary index of control-related neural engagement. Under the Go condition, group differences in theta power were limited and spatially inconsistent. Although a few posterior channels showed uncorrected differences, the Go condition did not reveal a robust or coherent pattern of increased theta recruitment in the AS group (see Supplementary S6 Table).

**Fig 6:**
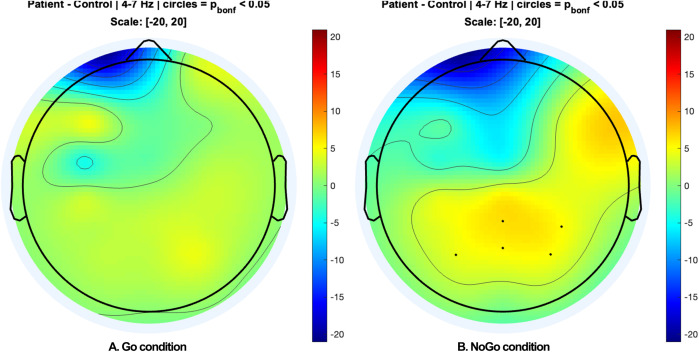
Topographic maps of theta power differences (4–7 Hz, 400–600 ms) between AS patients and healthy controls. (A) Go condition: Scalp distribution of theta power difference (Patient – Controls) in the 400–600 ms time window. No channels remained significant after Bonferroni correction. (B) NoGo condition: scalp distribution of theta power difference (Patient−Controls) in the 400–600 ms time window. The color bar represents the difference in theta power (arbitrary units). Warmer colors (yellow-red) indicate higher theta power in AS patients relative to controls, while cooler colors (blue) indicate lower theta power. Black dots indicate electrode sites with Bonferroni-corrected significance (p_bonf_ < 0.05).

By contrast, the NoGo condition revealed a more consistent enhancement of theta power in AS patients relative to controls, particularly over centro-parietal regions. Channel-wise comparisons indicated stronger theta activity in the AS group at multiple posterior and midline sites. After Bonferroni correction across the 26 tested channels, significant group differences remained at CPz, CP4, P3, Pz, and P4 (see Supplementary S7 Table, Fig 6). This pattern indicates that inhibitory processing in AS was accompanied by greater post-stimulus theta recruitment, especially over centro-parietal regions, consistent with heightened neural engagement during successful response withholding.

Importantly, several of these theta-enhanced sites overlapped spatially with electrodes implicated in the ERP findings, particularly within the central-to-parietal midline range. Although theta was treated here as a complementary rather than primary neural index, the convergence between enhanced NoGo theta activity and altered NoGo-P3–behavior coupling supports the broader interpretation that inhibitory control in AS relies on a more effortful pattern of control-related neural recruitment.

## Discussion

The present study investigated inhibitory control in AS using a Go/NoGo paradigm combined with EEG, with particular emphasis on the relationship between neural activity and behavioral performance. Several findings emerged. First, at the behavioral level, patients with AS showed lower Go accuracy and longer Go reaction times, indicating reduced efficiency during response execution. In contrast, NoGo accuracy was not impaired and was numerically higher in the AS group. Second, ERP analyses showed that the most functionally informative effect was not a simple mean-amplitude difference, but a group-dependent association between NoGo-P3 activity and inhibitory accuracy. Specifically, NoGo accuracy was positively associated with NoGo-P3 amplitude in the AS group, and this association differed significantly from controls at FCz, with Cz showing a similar but non-significant trend. Third, complementary theta analyses revealed stronger post-stimulus theta activity during NoGo processing in AS, particularly over centro-parietal sites. Taken together, these findings suggest altered brain–behavior coupling during inhibitory control in AS, with the most robust evidence emerging from the NoGo-P3 component.

The behavioral pattern is noteworthy because it does not conform to a simple global-deficit account. Patients with AS were slower and less accurate on Go trials, suggesting reduced efficiency in response execution. However, they did not show poorer NoGo accuracy. This dissociation implies that executive alterations in AS may be task-dependent rather than uniformly impaired across all response demands. Considering that patients with AS are often accompanied by fatigue, chronic pain, sleep disturbances, and an overall disease burden, these factors themselves may increase cognitive load and affect executive-control-related processing [13,18]. One plausible interpretation is that the AS group required greater control-related neural recruitment in order to maintain inhibitory performance, even while showing reduced efficiency in simpler execution-related processing. Importantly, the present data do not demonstrate compensation in a definitive causal sense; rather, they are consistent with a compensatory interpretation in which preserved or relatively maintained inhibitory accuracy is accompanied by altered neural engagement.

Within the ERP findings, the NoGo-P3 proved to be more informative than the N2. The N2 time window was examined because it remains a widely used index of early inhibitory and conflict-related processing [21–22]. However, in the present dataset, N2 effects were modest and less stable across electrodes. In addition, because N2 was quantified as mean amplitude within a predefined latency window rather than as individual peak negativity, its absolute values were not necessarily negative across all electrodes and conditions. Accordingly, the present findings do not support strong conclusions regarding early-stage inhibitory abnormalities in AS. By contrast, the later NoGo-P3 showed a clearer and more functionally meaningful association with behavioral performance. Previous studies have generally suggested that the later P3 component in Go/NoGo tasks is closely related to inhibitory evaluation, response monitoring, and the allocation of control-related resources [19,22,23,25]. More recent ERP research on NoGo tasks has further emphasized that later-stage P3-related activity tends to reflect successful inhibition and subsequent control evaluation more consistently than the N2 [26]. Therefore, in the present study, the NoGo-P3 is better suited to serve as the core neural index of altered inhibitory control in AS.

The most important result of the study lies in the altered coupling between NoGo-P3 amplitude and inhibitory accuracy. In the AS group, larger NoGo-P3 amplitudes were associated with better NoGo accuracy, whereas this relationship was not observed in controls. Critically, direct between-group comparison using the ERP × Group interaction confirmed that this association differed significantly at FCz. This point is important conceptually. If the analysis had been limited to mean group differences alone, the central finding of the study would have been much less clear. Instead, the present results suggest that the functional significance of the neural response differs across groups: in AS, successful inhibition appears to depend more strongly on the magnitude of late control-related neural engagement. In this sense, the altered pattern in AS may be better understood as a change in the brain–behavior relationship than as a simple increase or decrease in ERP amplitude. Previous research on healthy aging and mild cognitive impairment has also suggested that when differences in mean behavioral performance are limited, changes in the coupling pattern between neural activity and behavioral success may reveal underlying differences in regulatory strategies more effectively than mean-level differences alone [30,31]. This perspective is also applicable to understanding the task-dependent pattern of neural regulation observed in AS patients in the present study.

The topography of this effect also deserves attention. The most robust coupling difference was observed at FCz, with Cz showing a similar directional tendency. This fronto-central emphasis is consistent with the interpretation of the late NoGo component as a NoGo-P3 rather than canonical parietal P3b. In the context of the Go/NoGo paradigm, a fronto-central to centro-parietal late positive component is commonly interpreted as reflecting later-stage inhibitory evaluation, response monitoring, and control allocation [19,22,32]. Therefore, the present findings suggest that AS-related neural alterations are most clearly manifested at the later stage of control-related processing during successful inhibition, rather than at the stage of early conflict detection. This is also consistent with the pattern observed in our group-level comparisons: the N2 findings were less robust, whereas the functional relevance of the NoGo-P3 was more prominent.

The theta findings provide convergent, although secondary, support for this interpretation. In the NoGo condition, AS patients showed stronger theta power than controls, particularly over centro-parietal sites, and several of these effects survived Bonferroni correction. In response inhibition and cognitive control tasks, increased theta activity is often regarded as an index of greater recruitment of control-related neural resources [27–29]. Therefore, the enhancement of NoGo-related theta power observed in the present study may reflect greater oscillatory neural recruitment during inhibitory processing in patients with AS. However, this finding should still be interpreted with caution. In the present study, theta was analyzed as a complementary neural index, and no additional baseline normalization was applied to theta power beyond the standard ERP preprocessing procedures. Therefore, the theta findings are better understood as evidence supporting altered control-related neural engagement, rather than as independent mechanistic proof.

A clinically relevant implication of the present findings is that executive alteration in AS may not always manifest as overt behavioral failure. Instead, patients may achieve comparable or even numerically better inhibitory accuracy through a different and potentially more effortful pattern of neural recruitment. This is important because it suggests that average performance alone may underestimate clinically meaningful changes in neural control processes. From this perspective, the NoGo-P3—particularly at FCz—may represent a candidate electrophysiological marker of altered executive processing in AS. However, the present data do not justify describing it as a validated biomarker, and further replication in larger and clinically better-characterized samples will be necessary.In recent years, commentaries and cohort studies in rheumatology have also begun to emphasize that cognitive changes may constitute an independent and clinically meaningful dimension of disease burden in inflammatory rheumatic disorders, and that their manifestations are often intertwined with disease activity, affective symptoms, and overall functional status. This also supports the need for a more cautious interpretation of the present findings [7,19].

Several limitations should be acknowledged. First, the study employed a cross-sectional design, which precludes causal inference regarding the developmental or disease-related origin of the observed neural pattern. Second, the sample size was modest, especially for subgroup-level brain–behavior analyses. Third, the number of accepted NoGo epochs after preprocessing was relatively limited in the AS group, which may have reduced the stability of ERP and theta estimates. One patient was excluded from the NoGo ERP–behavior analyses because no artifact-free NoGo trials remained after preprocessing. Fourth, clinical characterization in the present study was limited: participants were classified on the basis of clinical diagnosis, but detailed measures of disease activity, inflammatory burden, pain, fatigue, sleep, mood, and medication status were not systematically incorporated into the analyses. As a result, the present findings should not be interpreted as direct evidence for a specific inflammation-to-brain mechanism. Although previous studies on inflammation, brain atrophy, and cognitive decline provide important background for this line of inquiry, in the absence of individualized inflammatory markers and longitudinal data, the present findings should not be directly interpreted as evidence for a specific mechanistic pathway [3,15,16,33]. Finally, theta power was analyzed within a predefined post-stimulus window without additional baseline normalization, and therefore should be interpreted conservatively.

Despite these limitations, the present study contributes to the emerging literature on cognition in AS by showing that inhibitory-control-related neural processing is altered in ways that are not fully captured by mean behavioral or ERP amplitude differences alone. The results highlight the value of brain–behavior coupling analysis in clinical cognitive neuroscience and suggest that the most meaningful neural alteration in AS may lie in how strongly successful inhibition depends on late control-related neural recruitment. Future studies with larger samples, more detailed clinical phenotyping, and longitudinal designs will be needed to determine whether this altered coupling pattern is stable across disease stages and whether it relates to clinically meaningful outcomes.

In summary, the present findings indicate that AS is associated with altered neural engagement during inhibitory control, with the clearest evidence emerging from the NoGo-P3 component and its relationship to behavioral performance. Rather than supporting a simple global-deficit model, the data suggest a more nuanced pattern in which successful inhibition in AS is accompanied by altered, and potentially more effortful, control-related neural recruitment.

## Supporting information

S1 Table Accepted epoch counts after preprocessing by group and condition. (XLSX)

S2 Table Comparison of mean ERP component amplitudes between groups in the Go condition. (XLSX)

S3 Table Comparison of mean ERP component amplitudes between groups in the NoGo condition. (XLSX)

S4 Table Brain–behavior correlation analyses of ERP metrics and task performance in AS patients and healthy controls. (XLSX)

S5 Table Correlation and interaction analyses for the association between NoGo-P3 amplitude and NoGo accuracy at FCz and Cz. (XLSX)

S6 Table Channel-wise theta power differences between AS patients and healthy controls in the Go condition, with Bonferroni-corrected results. (XLSX)

S7 Table Channel-wise theta power differences between AS patients and healthy controls in the NoGo condition, with Bonferroni-corrected results. (XLSX)

S8 Data Processed behavioral and ERP data underlying the analyses reported in this study. (XLSX)
