# Supplementary material for: Altered brain–behavior coupling during inhibitory control in ankylosing spondylitis: ERP evidence from NoGo-P3 component
Source: PLoS One. 2026 Jun 12;21(6):e0351397. doi: 10.1371/journal.pone.0351397 (PMC13262838; doi:10.1371/journal.pone.0351397)
Supplement: Attachment — Submitted filename: Rebuttal Letter.docx [file pone.0351397.s009.docx]

**Response to Reviewers**

Manuscript ID: PONE-D-25-42878
Title: Altered Brain–Behavior Coupling during Inhibitory Control in Ankylosing Spondylitis: ERP Evidence from N2 and P3b Components

Dear Editor and Reviewer,

We sincerely thank the Academic Editor and Reviewer for their careful evaluation of our manuscript and for the constructive comments that have substantially improved the clarity, rigor, and interpretability of the study. We have revised the manuscript thoroughly in response to all comments. Below, we provide a point-by-point response detailing how each concern has been addressed. All changes are highlighted in the revised manuscript with tracked changes.

Reviewer #1

Comment 1: Group Differences and Statistical Reporting

The authors repeatedly note that while there are visually apparent trends in ERP waveforms and topographies, statistical tests show no significant group differences (all p-values > 0.10). This point requires more explicit discussion, particularly given the reliance on within-group correlations.

Response:
We thank the reviewer for this important observation. In the revised manuscript, we have explicitly clarified the conceptual distinction between group-level mean differences and brain–behavior coupling mechanisms, which form the core analytic framework of this study.

Specifically, we now state that the absence of significant between-group differences in mean ERP amplitudes does not indicate preserved neural function in AS. Rather, it suggests that compensatory regulation in AS is more likely expressed through task- and condition-specific brain–behavior coupling, rather than through gross group-average amplitude effects. This clarification has been incorporated into:

the opening paragraph of the Discussion, where we explicitly address the interpretation of non-significant group-level ERP contrasts; and the Conclusion, where we emphasize that compensatory mechanisms are inferred from dynamic coupling patterns rather than static amplitude differences. These revisions ensure that visually apparent trends are not overinterpreted and that the rationale for focusing on within-group associations is transparent and theoretically grounded.

Comment 2: Limited N2 Component Discussion

The abstract and introduction refer to the N2 component, but the results and discussion sections contain no relevant N2 findings, leaving a gap in the narrative.

Response:
We appreciate the reviewer’s careful attention to this issue. In response, we have revised the manuscript to explicitly address the role of the N2 component and to prevent potential narrative inconsistency.

In the revised Results section, we now explicitly state that N2 amplitudes did not show significant group differences or reliable brain–behavior associations. In the Discussion, we clarify that this absence of robust N2 effects suggests that early conflict detection processes may be relatively preserved in AS, whereas later-stage regulatory and compensatory processes—indexed by P3b amplitude and theta power coupling—play a more prominent role in task performance.

By explicitly acknowledging the null N2 findings and contextualizing them within the temporal hierarchy of executive control, the revised manuscript now provides a balanced and coherent account of both ERP components introduced in the Introduction.

Comment 3: Figure Clarity and Presentation

The accuracy of ERP waveforms and topographic maps could be improved. Font sizes are small, and color graduations are not uniform across figures.

Response:
We thank the reviewer for this technical recommendation. All figures (Figures 1–6) have been revised to improve clarity and consistency. Specifically, we have:

re-exported all figures as high-resolution TIFF files in accordance with PLOS ONE guidelines;

increased font sizes for axes, labels, and colorbars to ensure readability;

standardized color scales across comparable topographic maps to facilitate visual comparison; and

removed unnecessary electrode markers to improve visual clarity while preserving interpretability.

These changes enhance the readability and presentation quality of the figures without altering the underlying data or statistical results.

Comment 4: Contextualization of the “Slower-but-Safer” Strategy

The authors describe a slower-but-safer strategy but do not sufficiently discuss how this compromise may be specific to AS.

Response:
We appreciate this insightful comment. In the revised manuscript, we have expanded the behavioral interpretation to more explicitly link the “slower-but-safer” strategy to the clinical characteristics of ankylosing spondylitis.

In the Behavioral Evidence subsection of the Discussion, we now discuss how chronic inflammation, fatigue, and pain—core features of AS—may constrain baseline cognitive efficiency and promote a strategic shift toward accuracy preservation under high-demand conditions. This behavioral adaptation is explicitly framed within the speed–accuracy trade-off framework and interpreted as an adaptive, albeit high-cost, compensatory response to limited cognitive resources in AS.

Additional Editorial Requirements

Formatting and file naming: The manuscript has been revised to comply with PLOS ONE style requirements and file-naming conventions.

Role of the funder: As clarified in the cover letter, the funders had no role in study design, data collection and analysis, decision to publish, or preparation of the manuscript.

ORCID: The corresponding author’s ORCID iD has been validated in Editorial Manager.

References: The reference list has been carefully reviewed. No retracted articles are cited, and all references are complete and up to date.

Closing Statement

We are grateful to the Academic Editor and Reviewer for their thoughtful and constructive feedback. We believe that the revisions have substantially strengthened the manuscript by clarifying key conceptual points, improving figure presentation, and providing a more balanced interpretation of the ERP findings. We hope that the revised manuscript is now suitable for publication in PLOS ONE.

Sincerely,

Lei Zhang
