# Supplementary material for: Altered brain–behavior coupling during inhibitory control in ankylosing spondylitis: ERP evidence from NoGo-P3 component
Source: PLoS One. 2026 Jun 12;21(6):e0351397. doi: 10.1371/journal.pone.0351397 (PMC13262838; doi:10.1371/journal.pone.0351397)
Supplement: Attachment — Submitted filename: Reviewer.docx [file pone.0351397.s010.docx]

**Reviewer #1 Report**

**Manuscript title:** *Altered Brain–Behavior Coupling during Inhibitory Control in Ankylosing Spondylitis: ERP Evidence from N2 and P3b Components*

**Recommendation: Major Revision**

**Confidential comments to the Editor**

This manuscript addresses a clinically relevant and under-explored topic—executive control alterations in ankylosing spondylitis (AS)—using a Go/NoGo ERP design and a brain–behavior coupling framework. The central idea (task-contingent coupling in AS, potentially consistent with compensatory recruitment) is interesting and could be suitable for a high-impact outlet.

However, the current submission has important methodological and inferential weaknesses that preclude acceptance in its present form: (i) insufficient control of multiple comparisons across ERP, time–frequency, and correlation analyses; (ii) questionable component labeling/interpretation of “P3b” given the topography and condition; (iii) reliance on “significant in one group but not the other” without formal group-difference tests; and (iv) potentially low NoGo trial counts after artifact rejection. These issues are addressable, but require substantial re-analysis and reframing.

**Comments to the Authors**

**1) Summary of the work and contribution**

The authors compare male AS patients (n=16) and age-matched controls (n=23) on a Go/NoGo task with 32-channel EEG. They focus on N2 (200–300 ms), a late positivity labeled “P3b” (400–600 ms), and theta-band power (4–7 Hz) within the 400–600 ms window. Behaviorally, AS participants show slower responses and reduced Go accuracy, with a trend toward higher NoGo accuracy. The main claim is that NoGo accuracy is coupled with late ERP amplitude at FCz/Cz in AS but not in controls, accompanied by enhanced theta power during inhibition, interpreted as compensatory control recruitment.

The topic is timely and the brain–behavior coupling angle is potentially valuable. Nevertheless, several core claims are not yet supported at the evidentiary standard expected for a Q1 journal.

**Major concerns (must be addressed)**

**2) Component identification: the “P3b” label is likely not justified**

The manuscript interprets a 400–600 ms positivity as **P3b**, yet the reported coupling is maximal at **FCz/Cz**, and the condition of interest is **NoGo** inhibition. In the Go/NoGo literature, the NoGo-related late positivity often has a more **fronto-central** distribution (frequently discussed as **NoGo-P3** and/or P3a-like control engagement), whereas canonical **P3b** is typically **centro-parietal** (often maximal at Pz/CPz) and linked to context updating.

**Requested actions:**

- Provide quantitative justification for labeling the component as P3b (e.g., scalp distribution of peak/mean activity; demonstrate parietal maximum if claiming P3b).
- Alternatively (and likely more appropriate), **relabel** the effect as **NoGo-P3** (or clarify P3a vs P3b distinction) and update the title/abstract accordingly.
- Include a figure showing scalp topography in the relevant window for each condition/group, and report where the component peaks.

**3) Multiple comparisons: correction strategy is currently insufficient/unclear**

The analysis includes many statistical tests (ERP across electrodes and conditions; numerous correlations; theta across many channels). The manuscript and supplement currently mix uncorrected p-values with statements implying correction (e.g., Bonferroni in the figure caption) without a coherent family-wise strategy.

**Requested actions:**

- Define families of tests and apply appropriate correction (e.g., FDR or Bonferroni) **consistently** for:
  1. ERP group comparisons (components × electrodes × conditions),
  2. Brain–behavior correlation matrices,
  3. Theta topography/channel tests.
- Consider a more Q1-appropriate approach such as:
  1. a priori ROI analyses (confirmatory) + corrected exploratory analyses, and/or
  2. mixed ANOVA / linear mixed modeling (Group × Condition × Electrode) for ERP, and/or
  3. cluster-based permutation testing for time–frequency topographies.

**Important:** Several key inferences (e.g., Cz correlation p=.035) may not survive correction; conclusions must match corrected results.

**4) “Coupling present in AS but absent in controls” requires a formal group-difference test**

The manuscript repeatedly interprets significance in AS and non-significance in controls as evidence of group differences. This is not statistically valid without directly testing whether the correlations differ between groups.

**Requested actions:**

- Compare correlation coefficients between groups using Fisher r-to-z, or
- Fit an interaction model: Accuracy ~ ERP + Group + ERP×Group (robust SEs recommended).
- Report the **interaction p-value** as the basis for claims of differential coupling.

**5) N2 polarity and measurement: mismatch between narrative and reported values**

The manuscript describes a “pronounced negative deflection” but several N2 means are positive in the tables, raising concerns about measurement choice, referencing, and/or window definition.

**Requested actions:**

- Clarify reference montage and polarity conventions.
- Confirm whether the N2 window (200–300 ms) truly captures the negative peak for each subject/condition.
- Consider reporting peak negativity or NoGo–Go difference waves for N2 if appropriate.
- Ensure waveforms include a visible zero-line and baseline.

**6) Trial numbers: NoGo trial count is likely low and may undermine ERP/theta reliability**

With 150 trials and a 4:1 Go:NoGo ratio, there are ~30 NoGo trials before rejection. After artifact rejection, NoGo epochs may be too few for stable ERP/time–frequency estimates.

**Requested actions:**

- Report, for each group, mean±SD and range of accepted Go and NoGo epochs after preprocessing.
- State any minimum-epoch exclusion threshold (e.g., exclude subjects with <15–20 clean NoGo trials).
- Consider reporting reliability checks (split-half) or at minimum discuss signal stability limitations.

**7) Clinical characterization and confounds are insufficient for mechanistic interpretation**

The discussion makes inflammation-to-brain mechanistic claims, but the paper does not report key clinical variables (e.g., disease activity indices, CRP/ESR, pain, fatigue, sleep, mood, medication/biologic use).

**Requested actions:**

- Provide basic clinical descriptors and medication status.
- If available, test whether the proposed neural markers relate to clinical measures (even exploratory).
- If not available, substantially temper mechanistic language and clearly acknowledge confounding factors.

**8) Time–frequency (theta) method reporting is too sparse**

The theta analysis is described as short-time FFT, but key parameters and normalization details are missing.

**Requested actions:**

- Specify window length, tapering, step size, frequency resolution, and baseline normalization method (dB vs percent change).
- Justify why FFT is appropriate for theta time-locking in this context or consider standard wavelet/multitaper approaches.
- Apply and report multiple-comparison correction across channels (or use cluster-based permutation).

**9) Overstatement of “compensation” and “biomarker” claims**

Given cross-sectional design, small sample, and correlation-based inference, the manuscript should not claim strong evidence for compensation or call the effect a biomarker without validation.

**Requested actions:**

- Rephrase to “consistent with compensatory recruitment” and “candidate marker.”
- Add a limitations paragraph emphasizing cross-sectional design, small n, trial count, and confounds.

**10) Recruitment status statement is problematic**

The manuscript states recruitment is ongoing “at the time of manuscript preparation.” For a submitted study, the analyzed sample should be clearly defined with a fixed cutoff.

**Requested actions:**

- Remove/replace with a clear recruitment period and final sample cutoff date.
- If this is an interim analysis, that must be explicitly justified (ideally preregistered), which is uncommon for this type of paper.

**Minor comments / presentation**

1. Correct typographical issues (e.g., “fRMI” → fMRI; “Yanchen” likely “Yancheng”; “a important role” → “an important role”).
2. Provide descriptive statistics (mean±SD) for behavioral variables alongside inferential tests.
3. Ensure figure captions match statistical procedures (e.g., if Bonferroni is claimed, show corrected p-values or explicitly mark corrected sites).
4. Clarify task details: practice trials, handedness, stimulus mapping, randomization, feedback, and whether ISI is jittered.
5. Consider reducing emphasis on N2 in the title if N2 is not a central supported finding after corrected analysis.

**Overall conclusion**

The study has promise, but substantial re-analysis and reframing are required. In particular, component identification, trial-count transparency, corrected inference, and proper tests of group differences in coupling are essential for publication in a Q1 venue.
