# Supplementary material for: Altered brain–behavior coupling during inhibitory control in ankylosing spondylitis: ERP evidence from NoGo-P3 component
Source: PLoS One. 2026 Jun 12;21(6):e0351397. doi: 10.1371/journal.pone.0351397 (PMC13262838; doi:10.1371/journal.pone.0351397)
Supplement: Attachment — Submitted filename: Response to Reviewer.docx [file pone.0351397.s011.docx]

**Dear Reviewer,**

We sincerely thank you for your careful reading of our manuscript and for your constructive comments. Your suggestions have helped us substantially improve the clarity, methodological transparency, and interpretive rigor of the manuscript. In the revised version, we have carefully addressed the major concerns by:
(1) directly testing between-group differences in brain–behavior coupling using ERP × Group interaction models;
(2) relabeling the later positive component in the NoGo condition as NoGo-P3 throughout the manuscript;
(3) clarifying that ERP amplitudes were quantified as mean amplitudes within predefined time windows;
(4) expanding the description of the theta analysis, including frequency range, time window, FFT parameters, and Bonferroni correction;
(5) reporting accepted epoch counts after preprocessing and clarifying the exclusion of one patient from NoGo ERP–behavior analyses due to the absence of artifact-free NoGo trials;
(6) moderating overly strong mechanistic interpretations.

Our point-by-point responses are provided below.

**Comment 1. A significant correlation in one group and a non-significant correlation in the other do not by themselves demonstrate a between-group difference.**

**Response:**
We agree. In the revised manuscript, we no longer rely on this contrast alone. Instead, we directly tested between-group differences in the NoGo-P3–behavior relationship using linear regression models including the ERP × Group interaction term. These analyses showed a significant interaction at FCz, whereas Cz showed a similar but non-significant trend. We revised the Results and Discussion accordingly and now present FCz as the most robust site of group-dependent brain–behavior coupling. Detailed correlation, Fisher’s r-to-z, and interaction results are reported in the Supplementary Material.

**Comment 2. The later positive component under the NoGo condition is more appropriately described as a NoGo-P3 rather than P3b.**

**Response:**
We agree and have revised the terminology throughout the manuscript. The later positive component under the NoGo condition is now consistently referred to as NoGo-P3 in the title, abstract, main text, figures, axis labels, and figure captions. This change better reflects both the task context and the functional interpretation of the component.

**Comment 3. The title and overall emphasis on N2 are too strong relative to the actual findings.**

**Response:**
The revised title now emphasizes the NoGo-P3 component, which better reflects the strongest and most informative finding of the study. N2 remains reported as an additional index of early conflict-related processing, but it is no longer framed as a central result.

**Comment 4. The ERP measurement approach should be clarified, especially because N2 values are not always negative.**

**Response:**
We have clarified this point in the revised Methods and Results. Both N2 and NoGo-P3 were quantified as mean amplitudes within predefined latency windows, rather than as peak amplitudes. Therefore, values within the N2 window were not necessarily negative across all electrodes and conditions, even when the waveform showed a negative-going deflection. We now explain this explicitly and interpret the N2 findings more cautiously.

**Comment 5. The theta analysis requires fuller methodological reporting.**

**Response:**
We have expanded the theta-analysis description in the revised manuscript. Theta power was analyzed in the 4–7 Hz range within the 400–600 ms post-stimulus window using FFT-based spectral estimation, with a sampling rate of 1000 Hz and NFFT = 1024. We also clarify that the primary analyses focused on five a priori midline electrodes, while the broader 26-channel topographical comparisons were exploratory and are reported in the Supplementary Material. No additional theta baseline normalization was applied beyond the standard preprocessing baseline correction.

**Comment 6. If Bonferroni correction was applied, the corrected results should be clearly reported.**

**Response:**
We agree. In the revised manuscript, Bonferroni correction is now reported explicitly for the exploratory channel-wise theta analyses. The Supplementary Material now provides the corrected channel-wise results for both the Go and NoGo conditions. In the Go condition, no channel remained significant after correction. In the NoGo condition, significant differences remained at CPz, CP4, P3, Pz, and P4 after Bonferroni correction. Figure captions were also revised so that the meaning of the significance markers is clear.

**Comment 7. Trial counts and data quality should be reported more transparently.**

**Response:**
We have added a more transparent description of data quality. Accepted epoch counts after preprocessing are now reported separately for Go and NoGo trials in each group, including mean values and ranges. We also explicitly state that one patient was excluded from NoGo ERP–behavior analyses because no artifact-free NoGo trials remained after preprocessing. These data are also summarized in the Supplementary Material.

**Comment 8. Behavioral descriptive statistics should be reported alongside inferential statistics.**

**Response:**
We agree and revised the behavioral Results accordingly. The manuscript now reports mean ± SD values for the main behavioral measures, including Go accuracy, Go reaction time, and NoGo accuracy. The behavioral figure and text were also revised so that descriptive and inferential results are reported consistently.

**Comment 9. Task details were insufficiently described.**

**Response:**
We have expanded the task description in the revised Methods section. The manuscript now clearly states that all participants were right-handed; that the Go stimulus was “W” and the NoGo stimulus was “M”; that participants pressed the “J” key for Go trials and withheld responses for NoGo trials; that the sequence was randomized; that no trial-by-trial feedback was provided during the formal task; that participants completed practice trials and proceeded only after reaching 100% accuracy; and that the ISI was fixed at 1000 ms.

**Comment 10. The interpretation is too mechanistic given the limited clinical characterization.**

**Response:**
We agree and have substantially moderated the interpretation. Group classification in the present study was based on clinical diagnosis, rather than detailed disease-activity or biomarker profiling. We now explicitly acknowledge that disease activity, inflammatory burden, pain, fatigue, sleep, mood, and medication status were not systematically incorporated into the analyses. Accordingly, the revised manuscript avoids implying a direct inflammation-to-brain pathway and instead frames the findings more cautiously as evidence of altered inhibitory-control-related neural processing in AS.

**Comment 11. Claims regarding “compensation” or “biomarker” are too strong.**

**Response:**
We revised the wording throughout the manuscript. We no longer describe the findings as direct proof of compensation or as an established biomarker. Instead, we state that the observed pattern is consistent with a compensatory interpretation and that the NoGo-P3, particularly at FCz, may represent a candidate electrophysiological marker of altered executive processing in AS.

**Comment 12. Wording implying ongoing recruitment is inappropriate.**

**Response:**
We have corrected this point. The revised manuscript now states that participants were recruited through August 2025, and that the present analyses were based on the final eligible sample.

**Comment 13. Figure labels, captions, and statistical descriptions should be aligned.**

**Response:**
We have carefully revised the figures and captions for consistency with the revised analyses and terminology. In particular, we unified the use of NoGo-P3, clarified the interpretation of the theta topographies and significance markers, and aligned the figure legends with the actual statistical results reported in the main text and Supplementary Material.

**Comment 14. Minor language and presentation issues remain.**

**Response:**
We carefully revised the manuscript for clarity, internal consistency, and presentation. This included tightening the Results and Discussion sections, harmonizing terminology across the text and figures, and revising figure legends to make them more descriptive and less interpretive.

We sincerely thank you again for your thoughtful comments. We believe that the revisions have substantially improved the manuscript and hope that the revised version satisfactorily addresses your concerns.

Sincerely,

Zheng-Hong Yu
