# Supplementary material for: Altered brain–behavior coupling during inhibitory control in ankylosing spondylitis: ERP evidence from NoGo-P3 component
Source: PLoS One. 2026 Jun 12;21(6):e0351397. doi: 10.1371/journal.pone.0351397 (PMC13262838; doi:10.1371/journal.pone.0351397)
Supplement: Peer review history

# Peer review: Decision Letter 0

**DOI:** 10.1371/journal.pone.0351397.r001

**Authors:** Stéphane Charpier

17 Dec 2025

PONE-D-25-42878

Altered Brain–Behavior Coupling during Inhibitory Control in Ankylosing Spondylitis: ERP Evidence from N2 and P3b Components

PLOS One

Dear Dr. Yu,

Thank you for submitting your manuscript to PLOS ONE. After careful consideration, we feel that it has merit but does not fully meet PLOS ONE’s publication criteria as it currently stands. Therefore, we invite you to submit a revised version of the manuscript that addresses the points raised during the review process.

Please submit your revised manuscript by Jan 31 2026 11:59PM. If you will need more time than this to complete your revisions, please reply to this message or contact the journal office at plosone@plos.org. When you're ready to submit your revision, log on to https://www.editorialmanager.com/pone/ and select the 'Submissions Needing Revision' folder to locate your manuscript file.

Please include the following items when submitting your revised manuscript:

- A rebuttal letter that responds to each point raised by the academic editor and reviewer(s). You should upload this letter as a separate file labeled 'Response to Reviewers'.
- A marked-up copy of your manuscript that highlights changes made to the original version. You should upload this as a separate file labeled 'Revised Manuscript with Track Changes'.
- An unmarked version of your revised paper without tracked changes. You should upload this as a separate file labeled 'Manuscript'.

If you would like to make changes to your financial disclosure, please include your updated statement in your cover letter. Guidelines for resubmitting your figure files are available below the reviewer comments at the end of this letter.

-->If applicable, we recommend that you deposit your laboratory protocols in protocols.io to enhance the reproducibility of your results. Protocols.io assigns your protocol its own identifier (DOI) so that it can be cited independently in the future. For instructions see: https://journals.plos.org/plosone/s/submission-guidelines#loc-laboratory-protocols. Additionally, PLOS ONE offers an option for publishing peer-reviewed Lab Protocol articles, which describe protocols hosted on protocols.io. Read more information on sharing protocols at https://plos.org/protocols?utm_medium=editorial-email&utm_source=authorletters&utm_campaign=protocols.

We look forward to receiving your revised manuscript.

Kind regards,

Stéphane Charpier

Academic Editor

PLOS One

**Journal Requirements:**

1. When submitting your revision, we need you to address these additional requirements.

-->Please ensure that your manuscript meets PLOS ONE's style requirements, including those for file naming. The PLOS ONE style templates can be found at

https://journals.plos.org/plosone/s/file?id=wjVg/PLOSOne_formatting_sample_main_body.pdf and

https://journals.plos.org/plosone/s/file?id=ba62/PLOSOne_formatting_sample_title_authors_affiliations.pdf

2. Thank you for stating the following financial disclosure:

This research was funded by General Project of Jiangsu Natural Science Foundation, grant number BK20221181

Please state what role the funders took in the study.  If the funders had no role, please state: "The funders had no role in study design, data collection and analysis, decision to publish, or preparation of the manuscript."

If this statement is not correct you must amend it as needed.

Please include this amended Role of Funder statement in your cover letter; we will change the online submission form on your behalf.

3. PLOS requires an ORCID iD for the corresponding author in Editorial Manager on papers submitted after December 6th, 2016. Please ensure that you have an ORCID iD and that it is validated in Editorial Manager. To do this, go to ‘Update my Information’ (in the upper left-hand corner of the main menu), and click on the Fetch/Validate link next to the ORCID field. This will take you to the ORCID site and allow you to create a new iD or authenticate a pre-existing iD in Editorial Manager.

4. Please upload a new copy of Figures 1 to 6, as the detail is not clear. Please follow the link for more information:  https://journals.plos.org/plosone/s/figures

5. If the reviewer comments include a recommendation to cite specific previously published works, please review and evaluate these publications to determine whether they are relevant and should be cited. There is no requirement to cite these works unless the editor has indicated otherwise.

6. Please review your reference list to ensure that it is complete and correct. If you have cited papers that have been retracted, please include the rationale for doing so in the manuscript text, or remove these references and replace them with relevant current references. Any changes to the reference list should be mentioned in the rebuttal letter that accompanies your revised manuscript. If you need to cite a retracted article, indicate the article’s retracted status in the References list and also include a citation and full reference for the retraction notice.

[Note: HTML markup is below. Please do not edit.]

Reviewers' comments:

Reviewer's Responses to Questions

**Comments to the Author**

1. Is the manuscript technically sound, and do the data support the conclusions?

The manuscript must describe a technically sound piece of scientific research with data that supports the conclusions. Experiments must have been conducted rigorously, with appropriate controls, replication, and sample sizes. The conclusions must be drawn appropriately based on the data presented.

Reviewer #1: Yes

**********

2. Has the statistical analysis been performed appropriately and rigorously?

Reviewer #1: Yes

**********

3. Have the authors made all data underlying the findings in their manuscript fully available?

The PLOS Data policy requires authors to make all data underlying the findings described in their manuscript fully available without restriction, with rare exception (please refer to the Data Availability Statement in the manuscript PDF file). The data should be provided as part of the manuscript or its supporting information, or deposited to a public repository. For example, in addition to summary statistics, the data points behind means, medians and variance measures should be available. If there are restrictions on publicly sharing data—e.g. participant privacy or use of data from a third party—those must be specified.

Reviewer #1: Yes

**********

4. Is the manuscript presented in an intelligible fashion and written in standard English?

PLOS ONE does not copyedit accepted manuscripts, so the language in submitted articles must be clear, correct, and unambiguous. Any typographical or grammatical errors should be corrected at revision, so please note any specific errors here.

Reviewer #1: Yes

**********

5. Review Comments to the Author

Please use the space provided to explain your answers to the questions above. You may also include additional comments for the author, including concerns about dual publication, research ethics, or publication ethics. (Please upload your review as an attachment if it exceeds 20,000 characters)

**Reviewer #1:**

This study makes a compelling case for a nuanced understanding of the cognitive impairment associated with ankylosing spondylitis (AS). The authors go beyond a simple deficit model and propose a compensation mechanism, which is an important and novel contribution. The use of event-related potentials (ERPs) and time frequency analysis provides a reliable neurophysiological basis for their behavioral findings.

Here is a detailed analysis of the text, outlining its strengths and areas for improvement.

Strengths

• Novelty and clinical relevance: The main hypothesis of the study, which indicates that cognitive changes in AS reflect compensatory processes and not simple deficits, is very novel and clinically relevant.

• Integrated Multi-modal Analysis: A combination of behavioral data (time and accuracy of response), analysis of ERP components (N2 and P3b) and time frequency analysis provides a powerful multi-modal approach. The convergence of these findings - where behavioral, ERP, and oscillatory data all indicate a compensatory mechanism - is an important strength that supports the conclusions of the authors.

• Robust Brain-Behavior Coupling: The key finding of a strong and exclusive brain-behavioral association in patients with AS during NoGo is convincing. A specific relationship between P3b amplitude and inhibitory specificity provides a robust neural signature of this compensation process that is not present in healthy controls.

• Clear Behavioral Characterization: The authors effectively describe a strategy of a slower but safer response in patients with AS. By demonstrating that patients have a slower response time in both correct Go and wrong NoGo trials, they convincingly argue against simple motor impairment and for a more deliberate and effortful cognitive strategy.

Weaknesses and Recommendations

• Group Differences and Statistical Reporting: The authors repeatedly note that while there are visually apparent trends in ERP waveforms and topographies, statistical tests (t-tests) show no significant group differences (all p-values > 0.10). This is a critical point that requires more explicit discussion, especially since the core of the paper relies on within-group correlations.

• Limited N2 Component Discussion: The abstract and introduction refer to the N2 component, which is linked to Conflict Tracking. However, the results and discussion sections contain no relevant findings concerning N2. This leaves a gap in the narrative and may mislead readers who expect a more balanced account of the two strands.

• Figure Clarity and Presentation: Although the ERP waveforms (Figures 2, 4) and topographic maps (Figures 3, 5 and 6) are informative, their accuracy could be improved. The font size of the labels and the axes is very small and is difficult to read. Also, the color graduations on topographic maps are not uniform for all figures, which may be confusing.

• Contextualization of the "Slower-but-Safer" Strategy: The authors introduce a slower but safer strategy and align it with the principle of speed-accuracy trade-off. However, they do not discuss how that particular compromise may be specific to AS. Given the nature of chronic inflammatory diseases, it would be of great value to discuss in more detail how fatigue, pain and other symptoms might affect this cognitive strategy.

This manuscript is a high-quality study offering a new and convincing perspective on the cognitive impairment of ankylosing spondylitis. The methodology is sound and the findings are well supported by converging behavioral and neurophysiological evidence. The recommendations outlined above aim to improve the presentation of the manuscript, clarify key points and deepen its contextual discussion, ultimately making it a more impactful and accessible contribution to the field. I recommend acceptance pending a few minor revisions.

Respectfully yours,

Sobhan Khodadadi.

**********

6. PLOS authors have the option to publish the peer review history of their article (what does this mean?). If published, this will include your full peer review and any attached files.

If you choose “no”, your identity will remain anonymous but your review may still be made public.

**Do you want your identity to be public for this peer review?**   For information about this choice, including consent withdrawal, please see our Privacy Policy.

Reviewer #1: No

**********

[NOTE: If reviewer comments were submitted as an attachment file, they will be attached to this email and accessible via the submission site. Please log into your account, locate the manuscript record, and check for the action link "View Attachments". If this link does not appear, there are no attachment files.]

To ensure your figures meet our technical requirements, please review our figure guidelines: https://journals.plos.org/plosone/s/figures

You may also use PLOS’s free figure tool, NAAS, to help you prepare publication quality figures: https://journals.plos.org/plosone/s/figures#loc-tools-for-figure-preparation.

NAAS will assess whether your figures meet our technical requirements by comparing each figure against our figure specifications.

# Author comment: Author response to Decision Letter 1

**DOI:** 10.1371/journal.pone.0351397.r002

6 Jan 2026

All comments raised by the Academic Editor and the Reviewer have been addressed in detail in the rebuttal letter, which has been uploaded as a separate file.

Attachment Submitted filename: Rebuttal Letter.docx

# Peer review: Decision Letter 1

**DOI:** 10.1371/journal.pone.0351397.r003

**Authors:** John Falode

4 Mar 2026

PONE-D-25-42878R1

Altered Brain–Behavior Coupling during Inhibitory Control in Ankylosing Spondylitis: ERP Evidence from N2 and P3b Components

PLOS One

Dear Dr. Yu,

Thank you for submitting your manuscript to PLOS ONE. After careful consideration, we feel that it has merit but does not fully meet PLOS ONE’s publication criteria as it currently stands. Therefore, we invite you to submit a revised version of the manuscript that addresses the points raised during the review process.

Please submit your revised manuscript by Apr 18 2026 11:59PM. If you will need more time than this to complete your revisions, please reply to this message or contact the journal office at plosone@plos.org. When you're ready to submit your revision, log on to https://www.editorialmanager.com/pone/ and select the 'Submissions Needing Revision' folder to locate your manuscript file.

Please include the following items when submitting your revised manuscript:

- A letter that responds to each point raised by the academic editor and reviewer(s). You should upload this letter as a separate file labeled 'Response to Reviewers'.
- A marked-up copy of your manuscript that highlights changes made to the original version. You should upload this as a separate file labeled 'Revised Manuscript with Track Changes'.
- An unmarked version of your revised paper without tracked changes. You should upload this as a separate file labeled 'Manuscript'.

-->If you would like to make changes to your financial disclosure, please include your updated statement in your cover letter. Guidelines for resubmitting your figure files are available below the reviewer comments at the end of this letter.

If applicable, we recommend that you deposit your laboratory protocols in protocols.io to enhance the reproducibility of your results. Protocols.io assigns your protocol its own identifier (DOI) so that it can be cited independently in the future. For instructions see: https://journals.plos.org/plosone/s/submission-guidelines#loc-laboratory-protocols. Additionally, PLOS ONE offers an option for publishing peer-reviewed Lab Protocol articles, which describe protocols hosted on protocols.io. Read more information on sharing protocols at https://plos.org/protocols?utm_medium=editorial-email&utm_source=authorletters&utm_campaign=protocols.

We look forward to receiving your revised manuscript.

Kind regards,

John Adeolu Falode

Academic Editor

PLOS One

**Journal Requirements:**

1. If the reviewer comments include a recommendation to cite specific previously published works, please review and evaluate these publications to determine whether they are relevant and should be cited. There is no requirement to cite these works unless the editor has indicated otherwise.

2. Please review your reference list to ensure that it is complete and correct. If you have cited papers that have been retracted, please include the rationale for doing so in the manuscript text, or remove these references and replace them with relevant current references. Any changes to the reference list should be mentioned in the rebuttal letter that accompanies your revised manuscript. If you need to cite a retracted article, indicate the article’s retracted status in the References list and also include a citation and full reference for the retraction notice.

[Note: HTML markup is below. Please do not edit.]

Reviewers' comments:

Reviewer's Responses to Questions

**Comments to the Author**

1. If the authors have adequately addressed your comments raised in a previous round of review and you feel that this manuscript is now acceptable for publication, you may indicate that here to bypass the “Comments to the Author” section, enter your conflict of interest statement in the “Confidential to Editor” section, and submit your "Accept" recommendation.

Reviewer #2: (No Response)

**********

2. Is the manuscript technically sound, and do the data support the conclusions?

The manuscript must describe a technically sound piece of scientific research with data that supports the conclusions. Experiments must have been conducted rigorously, with appropriate controls, replication, and sample sizes. The conclusions must be drawn appropriately based on the data presented.

Reviewer #2: Yes

**********

3. Has the statistical analysis been performed appropriately and rigorously?

Reviewer #2: Yes

**********

4. Have the authors made all data underlying the findings in their manuscript fully available?

The PLOS Data policy requires authors to make all data underlying the findings described in their manuscript fully available without restriction, with rare exception (please refer to the Data Availability Statement in the manuscript PDF file). The data should be provided as part of the manuscript or its supporting information, or deposited to a public repository. For example, in addition to summary statistics, the data points behind means, medians and variance measures should be available. If there are restrictions on publicly sharing data—e.g. participant privacy or use of data from a third party—those must be specified.

Reviewer #2: Yes

**********

5. Is the manuscript presented in an intelligible fashion and written in standard English?

PLOS ONE does not copyedit accepted manuscripts, so the language in submitted articles must be clear, correct, and unambiguous. Any typographical or grammatical errors should be corrected at revision, so please note any specific errors here.

Reviewer #2: Yes

**********

6. Review Comments to the Author

Please use the space provided to explain your answers to the questions above. You may also include additional comments for the author, including concerns about dual publication, research ethics, or publication ethics. (Please upload your review as an attachment if it exceeds 20,000 characters)

**Reviewer #2:**   Reviewer #1 Report

Manuscript title: Altered Brain–Behavior Coupling during Inhibitory Control in Ankylosing Spondylitis: ERP Evidence from N2 and P3b Components

Recommendation: Major Revision

Confidential comments to the Editor

This manuscript addresses a clinically relevant and under-explored topic—executive control alterations in ankylosing spondylitis (AS)—using a Go/NoGo ERP design and a brain–behavior coupling framework. The central idea (task-contingent coupling in AS, potentially consistent with compensatory recruitment) is interesting and could be suitable for a high-impact outlet.

However, the current submission has important methodological and inferential weaknesses that preclude acceptance in its present form: (i) insufficient control of multiple comparisons across ERP, time–frequency, and correlation analyses; (ii) questionable component labeling/interpretation of “P3b” given the topography and condition; (iii) reliance on “significant in one group but not the other” without formal group-difference tests; and (iv) potentially low NoGo trial counts after artifact rejection. These issues are addressable, but require substantial re-analysis and reframing.

________________________________________

Comments to the Authors

1) Summary of the work and contribution

The authors compare male AS patients (n=16) and age-matched controls (n=23) on a Go/NoGo task with 32-channel EEG. They focus on N2 (200–300 ms), a late positivity labeled “P3b” (400–600 ms), and theta-band power (4–7 Hz) within the 400–600 ms window. Behaviorally, AS participants show slower responses and reduced Go accuracy, with a trend toward higher NoGo accuracy. The main claim is that NoGo accuracy is coupled with late ERP amplitude at FCz/Cz in AS but not in controls, accompanied by enhanced theta power during inhibition, interpreted as compensatory control recruitment.

The topic is timely and the brain–behavior coupling angle is potentially valuable. Nevertheless, several core claims are not yet supported at the evidentiary standard expected for a Q1 journal.

________________________________________

Major concerns (must be addressed)

2) Component identification: the “P3b” label is likely not justified

The manuscript interprets a 400–600 ms positivity as P3b, yet the reported coupling is maximal at FCz/Cz, and the condition of interest is NoGo inhibition. In the Go/NoGo literature, the NoGo-related late positivity often has a more fronto-central distribution (frequently discussed as NoGo-P3 and/or P3a-like control engagement), whereas canonical P3b is typically centro-parietal (often maximal at Pz/CPz) and linked to context updating.

Requested actions:

• Provide quantitative justification for labeling the component as P3b (e.g., scalp distribution of peak/mean activity; demonstrate parietal maximum if claiming P3b).

• Alternatively (and likely more appropriate), relabel the effect as NoGo-P3 (or clarify P3a vs P3b distinction) and update the title/abstract accordingly.

• Include a figure showing scalp topography in the relevant window for each condition/group, and report where the component peaks.

3) Multiple comparisons: correction strategy is currently insufficient/unclear

The analysis includes many statistical tests (ERP across electrodes and conditions; numerous correlations; theta across many channels). The manuscript and supplement currently mix uncorrected p-values with statements implying correction (e.g., Bonferroni in the figure caption) without a coherent family-wise strategy.

Requested actions:

• Define families of tests and apply appropriate correction (e.g., FDR or Bonferroni) consistently for:

1. ERP group comparisons (components × electrodes × conditions),

2. Brain–behavior correlation matrices,

3. Theta topography/channel tests.

• Consider a more Q1-appropriate approach such as:

o a priori ROI analyses (confirmatory) + corrected exploratory analyses, and/or

o mixed ANOVA / linear mixed modeling (Group × Condition × Electrode) for ERP, and/or

o cluster-based permutation testing for time–frequency topographies.

Important: Several key inferences (e.g., Cz correlation p=.035) may not survive correction; conclusions must match corrected results.

4) “Coupling present in AS but absent in controls” requires a formal group-difference test

The manuscript repeatedly interprets significance in AS and non-significance in controls as evidence of group differences. This is not statistically valid without directly testing whether the correlations differ between groups.

Requested actions:

• Compare correlation coefficients between groups using Fisher r-to-z, or

• Fit an interaction model: Accuracy ~ ERP + Group + ERP×Group (robust SEs recommended).

• Report the interaction p-value as the basis for claims of differential coupling.

5) N2 polarity and measurement: mismatch between narrative and reported values

The manuscript describes a “pronounced negative deflection” but several N2 means are positive in the tables, raising concerns about measurement choice, referencing, and/or window definition.

Requested actions:

• Clarify reference montage and polarity conventions.

• Confirm whether the N2 window (200–300 ms) truly captures the negative peak for each subject/condition.

• Consider reporting peak negativity or NoGo–Go difference waves for N2 if appropriate.

• Ensure waveforms include a visible zero-line and baseline.

6) Trial numbers: NoGo trial count is likely low and may undermine ERP/theta reliability

With 150 trials and a 4:1 Go:NoGo ratio, there are ~30 NoGo trials before rejection. After artifact rejection, NoGo epochs may be too few for stable ERP/time–frequency estimates.

Requested actions:

• Report, for each group, mean±SD and range of accepted Go and NoGo epochs after preprocessing.

• State any minimum-epoch exclusion threshold (e.g., exclude subjects with <15–20 clean NoGo trials).

• Consider reporting reliability checks (split-half) or at minimum discuss signal stability limitations.

7) Clinical characterization and confounds are insufficient for mechanistic interpretation

The discussion makes inflammation-to-brain mechanistic claims, but the paper does not report key clinical variables (e.g., disease activity indices, CRP/ESR, pain, fatigue, sleep, mood, medication/biologic use).

Requested actions:

• Provide basic clinical descriptors and medication status.

• If available, test whether the proposed neural markers relate to clinical measures (even exploratory).

• If not available, substantially temper mechanistic language and clearly acknowledge confounding factors.

8) Time–frequency (theta) method reporting is too sparse

The theta analysis is described as short-time FFT, but key parameters and normalization details are missing.

Requested actions:

• Specify window length, tapering, step size, frequency resolution, and baseline normalization method (dB vs percent change).

• Justify why FFT is appropriate for theta time-locking in this context or consider standard wavelet/multitaper approaches.

• Apply and report multiple-comparison correction across channels (or use cluster-based permutation).

9) Overstatement of “compensation” and “biomarker” claims

Given cross-sectional design, small sample, and correlation-based inference, the manuscript should not claim strong evidence for compensation or call the effect a biomarker without validation.

Requested actions:

• Rephrase to “consistent with compensatory recruitment” and “candidate marker.”

• Add a limitations paragraph emphasizing cross-sectional design, small n, trial count, and confounds.

10) Recruitment status statement is problematic

The manuscript states recruitment is ongoing “at the time of manuscript preparation.” For a submitted study, the analyzed sample should be clearly defined with a fixed cutoff.

Requested actions:

• Remove/replace with a clear recruitment period and final sample cutoff date.

• If this is an interim analysis, that must be explicitly justified (ideally preregistered), which is uncommon for this type of paper.

________________________________________

Minor comments / presentation

1. Correct typographical issues (e.g., “fRMI” → fMRI; “Yanchen” likely “Yancheng”; “a important role” → “an important role”).

2. Provide descriptive statistics (mean±SD) for behavioral variables alongside inferential tests.

3. Ensure figure captions match statistical procedures (e.g., if Bonferroni is claimed, show corrected p-values or explicitly mark corrected sites).

4. Clarify task details: practice trials, handedness, stimulus mapping, randomization, feedback, and whether ISI is jittered.

5. Consider reducing emphasis on N2 in the title if N2 is not a central supported finding after corrected analysis.

________________________________________

Overall conclusion

The study has promise, but substantial re-analysis and reframing are required. In particular, component identification, trial-count transparency, corrected inference, and proper tests of group differences in coupling are essential for publication in a Q1 venue.

**********

7. PLOS authors have the option to publish the peer review history of their article (what does this mean?). If published, this will include your full peer review and any attached files.

If you choose “no”, your identity will remain anonymous but your review may still be made public.

**Do you want your identity to be public for this peer review?**   For information about this choice, including consent withdrawal, please see our Privacy Policy.

Reviewer #2: **Yes:**   soomaayeh heysieattalab

**********

[NOTE: If reviewer comments were submitted as an attachment file, they will be attached to this email and accessible via the submission site. Please log into your account, locate the manuscript record, and check for the action link "View Attachments". If this link does not appear, there are no attachment files.]

To ensure your figures meet our technical requirements, please review our figure guidelines: https://journals.plos.org/plosone/s/figures

You may also use PLOS’s free figure tool, NAAS, to help you prepare publication quality figures: https://journals.plos.org/plosone/s/figures#loc-tools-for-figure-preparation.

NAAS will assess whether your figures meet our technical requirements by comparing each figure against our figure specifications.

Attachment Submitted filename: Reviewer.docx

# Author comment: Author response to Decision Letter 2

**DOI:** 10.1371/journal.pone.0351397.r004

18 Apr 2026

Dear Reviewer,

We sincerely thank you for your careful reading of our manuscript and for your constructive comments. Your suggestions have helped us substantially improve the clarity, methodological transparency, and interpretive rigor of the manuscript. In the revised version, we have carefully addressed the major concerns by:

(1) directly testing between-group differences in brain–behavior coupling using ERP × Group interaction models;

(2) relabeling the later positive component in the NoGo condition as NoGo-P3 throughout the manuscript;

(3) clarifying that ERP amplitudes were quantified as mean amplitudes within predefined time windows;

(4) expanding the description of the theta analysis, including frequency range, time window, FFT parameters, and Bonferroni correction;

(5) reporting accepted epoch counts after preprocessing and clarifying the exclusion of one patient from NoGo ERP–behavior analyses due to the absence of artifact-free NoGo trials;

(6) moderating overly strong mechanistic interpretations.

Our point-by-point responses are provided below.

Comment 1. A significant correlation in one group and a non-significant correlation in the other do not by themselves demonstrate a between-group difference.

Response:

We agree. In the revised manuscript, we no longer rely on this contrast alone. Instead, we directly tested between-group differences in the NoGo-P3–behavior relationship using linear regression models including the ERP × Group interaction term. These analyses showed a significant interaction at FCz, whereas Cz showed a similar but non-significant trend. We revised the Results and Discussion accordingly and now present FCz as the most robust site of group-dependent brain–behavior coupling. Detailed correlation, Fisher’s r-to-z, and interaction results are reported in the Supplementary Material.

Comment 2. The later positive component under the NoGo condition is more appropriately described as a NoGo-P3 rather than P3b.

Response:

We agree and have revised the terminology throughout the manuscript. The later positive component under the NoGo condition is now consistently referred to as NoGo-P3 in the title, abstract, main text, figures, axis labels, and figure captions. This change better reflects both the task context and the functional interpretation of the component.

Comment 3. The title and overall emphasis on N2 are too strong relative to the actual findings.

Response:

The revised title now emphasizes the NoGo-P3 component, which better reflects the strongest and most informative finding of the study. N2 remains reported as an additional index of early conflict-related processing, but it is no longer framed as a central result.

Comment 4. The ERP measurement approach should be clarified, especially because N2 values are not always negative.

Response:

We have clarified this point in the revised Methods and Results. Both N2 and NoGo-P3 were quantified as mean amplitudes within predefined latency windows, rather than as peak amplitudes. Therefore, values within the N2 window were not necessarily negative across all electrodes and conditions, even when the waveform showed a negative-going deflection. We now explain this explicitly and interpret the N2 findings more cautiously.

Comment 5. The theta analysis requires fuller methodological reporting.

Response:

We have expanded the theta-analysis description in the revised manuscript. Theta power was analyzed in the 4–7 Hz range within the 400–600 ms post-stimulus window using FFT-based spectral estimation, with a sampling rate of 1000 Hz and NFFT = 1024. We also clarify that the primary analyses focused on five a priori midline electrodes, while the broader 26-channel topographical comparisons were exploratory and are reported in the Supplementary Material. No additional theta baseline normalization was applied beyond the standard preprocessing baseline correction.

Comment 6. If Bonferroni correction was applied, the corrected results should be clearly reported.

Response:

We agree. In the revised manuscript, Bonferroni correction is now reported explicitly for the exploratory channel-wise theta analyses. The Supplementary Material now provides the corrected channel-wise results for both the Go and NoGo conditions. In the Go condition, no channel remained significant after correction. In the NoGo condition, significant differences remained at CPz, CP4, P3, Pz, and P4 after Bonferroni correction. Figure captions were also revised so that the meaning of the significance markers is clear.

Comment 7. Trial counts and data quality should be reported more transparently.

Response:

We have added a more transparent description of data quality. Accepted epoch counts after preprocessing are now reported separately for Go and NoGo trials in each group, including mean values and ranges. We also explicitly state that one patient was excluded from NoGo ERP–behavior analyses because no artifact-free NoGo trials remained after preprocessing. These data are also summarized in the Supplementary Material.

Comment 8. Behavioral descriptive statistics should be reported alongside inferential statistics.

Response:

We agree and revised the behavioral Results accordingly. The manuscript now reports mean ± SD values for the main behavioral measures, including Go accuracy, Go reaction time, and NoGo accuracy. The behavioral figure and text were also revised so that descriptive and inferential results are reported consistently.

Comment 9. Task details were insufficiently described.

Response:

We have expanded the task description in the revised Methods section. The manuscript now clearly states that all participants were right-handed; that the Go stimulus was “W” and the NoGo stimulus was “M”; that participants pressed the “J” key for Go trials and withheld responses for NoGo trials; that the sequence was randomized; that no trial-by-trial feedback was provided during the formal task; that participants completed practice trials and proceeded only after reaching 100% accuracy; and that the ISI was fixed at 1000 ms.

Comment 10. The interpretation is too mechanistic given the limited clinical characterization.

Response:

We agree and have substantially moderated the interpretation. Group classification in the present study was based on clinical diagnosis, rather than detailed disease-activity or biomarker profiling. We now explicitly acknowledge that disease activity, inflammatory burden, pain, fatigue, sleep, mood, and medication status were not systematically incorporated into the analyses. Accordingly, the revised manuscript avoids implying a direct inflammation-to-brain pathway and instead frames the findings more cautiously as evidence of altered inhibitory-control-related neural processing in AS.

Comment 11. Claims regarding “compensation” or “biomarker” are too strong.

Response:

We revised the wording throughout the manuscript. We no longer describe the findings as direct proof of compensation or as an established biomarker. Instead, we state that the observed pattern is consistent with a compensatory interpretation and that the NoGo-P3, particularly at FCz, may represent a candidate electrophysiological marker of altered executive processing in AS.

Comment 12. Wording implying ongoing recruitment is inappropriate.

Response:

We have corrected this point. The revised manuscript now states that participants were recruited through August 2025, and that the present analyses were based on the final eligible sample.

Comment 13. Figure labels, captions, and statistical descriptions should be aligned.

Response:

We have carefully revised the figures and captions for consistency with the revised analyses and terminology. In particular, we unified the use of NoGo-P3, clarified the interpretation of the theta topographies and significance markers, and aligned the figure legends with the actual statistical results reported in the main text and Supplementary Material.

Comment 14. Minor language and presentation issues remain.

Response:

We carefully revised the manuscript for clarity, internal consistency, and presentation. This included tightening the Results and Discussion sections, harmonizing terminology across the text and figures, and revising figure legends to make them more descriptive and less interpretive.

We sincerely thank you again for your thoughtful comments. We believe that the revisions have substantially improved the manuscript and hope that the revised version satisfactorily addresses your concerns.

Sincerely,

Zheng-Hong Yu

Attachment Submitted filename: Response to Reviewer.docx

# Editor report: Decision Letter 2

**DOI:** 10.1371/journal.pone.0351397.r005

**Authors:** John Falode

28 May 2026

Altered Brain–Behavior Coupling during Inhibitory Control in Ankylosing Spondylitis: ERP Evidence from NoGo-P3 Component

PONE-D-25-42878R2

Dear Dr. Yu,

We’re pleased to inform you that your manuscript has been judged scientifically suitable for publication and will be formally accepted for publication once it meets all outstanding technical requirements.

Within one week, you’ll receive an e-mail detailing the required amendments. When these have been addressed, you’ll receive a formal acceptance letter, and your manuscript will be scheduled for publication.

An invoice will be generated when your article is formally accepted. Please note, if your institution has a publishing partnership with PLOS and your article meets the relevant criteria, all or part of your publication costs will be covered. Please make sure your user information is up-to-date by logging into Editorial Manager at Editorial Manager® and clicking the ‘Update My Information' link at the top of the page. For questions related to billing, please contact billing support.

If your institution or institutions have a press office, please notify them about your upcoming paper to help maximize its impact. If they’ll be preparing press materials, please inform our press team as soon as possible -- no later than 48 hours after receiving the formal acceptance. Your manuscript will remain under strict press embargo until 2 pm Eastern Time on the date of publication. For more information, please contact onepress@plos.org.

Kind regards,

John Adeolu Falode

Academic Editor

PLOS One

Additional Editor Comments (optional):

Reviewers' comments:

# Editor report: Acceptance letter

**DOI:** 10.1371/journal.pone.0351397.r006

**Authors:** John Falode

PONE-D-25-42878R2

PLOS One

Dear Dr. Yu,

I'm pleased to inform you that your manuscript has been deemed suitable for publication in PLOS One. Congratulations! Your manuscript is now being handed over to our production team.

At this stage, our production department will prepare your paper for publication. This includes ensuring the following:

* All references, tables, and figures are properly cited

* All relevant supporting information is included in the manuscript submission,

* There are no issues that prevent the paper from being properly typeset

You will receive further instructions from the production team, including instructions on how to review your proof when it is ready. Please keep in mind that we are working through a large volume of accepted articles, so please give us a few days to review your paper and let you know the next and final steps.

Lastly, if your institution or institutions have a press office, please let them know about your upcoming paper now to help maximize its impact. If they'll be preparing press materials, please inform our press team within the next 48 hours. Your manuscript will remain under strict press embargo until 2 pm Eastern Time on the date of publication. For more information, please contact onepress@plos.org.

You will receive an invoice from PLOS for your publication fee after your manuscript has reached the completed accept phase. If you receive an email requesting payment before acceptance or for any other service, this may be a phishing scheme. Learn how to identify phishing emails and protect your accounts at https://explore.plos.org/phishing.

If we can help with anything else, please email us at customercare@plos.org.

Thank you for submitting your work to PLOS ONE and supporting open access.

Kind regards,

PLOS ONE Editorial Office Staff

on behalf of

Dr. John Adeolu Falode

Academic Editor

PLOS One
